# Personality traits and motivational patterns of social media use among undergraduate medical students at King Faisal University, Saudi Arabia

**DOI:** 10.3389/fpsyg.2026.1813278

**Published:** 2026-05-14

**Authors:** Ayoob Lone, Faisal Waleed Aloraifi, Joud Mohammed Almulla, Hassan Ali Almirza, Saad Habib Alotaibi, Yousif Mohamed Ahmed Elmosaad, Nawaf Al Khashram, Sayed Abdul Qadar Quadri, Naveed Gani

**Affiliations:** 1 Department of Clinical Neurosciences, College of Medicine, King Faisal University, Al-Ahsa, Saudi Arabia; 2 College of Medicine, King Faisal University, Al-Ahsa, Saudi Arabia; 3 Department of Public Health, College of Applied Medical Sciences, King Faisal University, Al-Ahsa, Saudi Arabia; 4 Department of Biomedical Sciences, College of Medicine, King Faisal University, Al-Ahsa, Saudi Arabia; 5 Department of Medical Education, College of Medicine, King Faisal University, Al-Ahsa, Saudi Arabia

**Keywords:** medical students, motivational pattern, personality traits, Saudi Arabia, social media

## Abstract

**Introduction:**

Social media has become deeply embedded in the daily lives of medical students, shaping communication, learning, and leisure activities. While demographic predictors of social media use are well documented, the role of personality traits in shaping distinct motivational patterns remains underexplored in the Saudi Arabian context.

**Aim and objectives:**

Drawing on the Big Five personality framework, this study examined how Big Five personality traits predict socialization, entertainment, informational, and academic motives for social media engagement among medical students.

**Methods:**

A cross-sectional survey was conducted among 370 undergraduate medical students at King Faisal University, Saudi Arabia. Participants completed the Big Five Inventory–10 and the Social Networking Usage Questionnaire. Hierarchical multiple regression analyses assessed the unique contribution of personality traits to social media motives after controlling for sociodemographic characteristics and patterns of social media engagement.

**Results:**

Extraversion and neuroticism were both positively associated with socialization (*β* = 0.17 and *β* = 0.15, respectively; *p* < 0.01) and entertainment motives (*β* = 0.16 for both; *p* < 0.01). Openness to experience predicted informational motives (*β* = 0.15, *p* < 0.01) but was not associated with academic use. Conscientiousness was positively associated with entertainment motives (*β* = 0.16, *p* < 0.01), while agreeableness did not significantly predict any motivational domain. Collectively, the Big Five traits explained a modest but statistically significant additional proportion of variance in overall social media motives beyond demographic and usage factors (Δ*R*^2^ = 0.03, *p* < 0.01).

**Conclusion:**

Personality traits exert a differentiated and meaningful influence on the motivational drivers of social media use among medical students. Extraversion and neuroticism primarily shape socially and emotionally oriented engagement, openness facilitates exploratory information-seeking, and conscientiousness supports regulated entertainment use. These findings underscore the value of personality-informed approaches in understanding digital behavior within medical education and may inform targeted interventions to promote adaptive social media engagement.

## Introduction

Social media and social networking sites are now deeply embedded into almost every aspect of daily life (Salloum et al., 2017; Dzogbenuku et al., 2022) and exert significant influence on the lives of users globally. According to STATISTA by October 2025, 5.66 billion or 68.7 per cent of the world’s total population are using social media (Statista, 2025). This global trend is reflected in the Kingdom of Saudi Arabia as well, where social media usage has seen an exponential rise. The number of users rose from 7.6 million in 2014 to 35.33 million in 2024, demonstrating the rapid expansion of social media platforms across the country (Gmi Blogger Saudi Arabia Social Media Statistics, 2024). While this remarkable growth has raised concerns regarding excessive or potentially harmful patterns of use among the Saudi population (Alrasheed et al., 2022; AlHamad and AlAmri, 2021; Beyari, 2023), the broader impact of social media remains a subject of ongoing debate. Evidence suggests that social media use is associated with potential risks, including problematic use and psychological distress, although findings remain mixed and context-dependent, with effects varying across patterns of engagement and individual differences (Appel et al., 2020; Huang, 2022; Kross et al., 2021; Orben, 2020). Within this context, there remains a need for nuanced research examining how and why individuals engage with social media, particularly among populations such as young adults, university students, and medical students, who are characterized by high levels of digital exposure.

Social media use has witnessed a sustained growth in recent times (Tandon et al., 2021). It has profoundly shaped human–internet interactions, influencing the ways individuals communicate, access information, and engage with digital content (Ahmed et al., 2019). Understanding the characteristics of frequent social media users is therefore of considerable interest to researchers and policymakers alike. Several studies have examined the demographic variables, such as age and gender of the social media user providing an insight into the patterns of engagement (Chidiac et al., 2022; Bonsaksen et al., 2024). Prior research consistently shows that social media usage tends to decline with increasing age, whereas males generally exhibit higher levels of activity and engagement compared to females (Chidiac et al., 2022; Alfaya et al., 2023). These patterns highlight the importance of considering demographic variations when studying social media behaviors and their potential impacts.

In the Saudi Arabian context, psychological factors, especially personality traits, are inadequately explored, and there is a paucity of empirical data covering this area. Despite growing theoretical and empirical evidence suggesting that personality characteristics are highly relevant for understanding online human behavior (Amichai-Hamburger, 2007), including patterns of social media use, this relationship has not been sufficiently examined within the cultural and societal framework of Saudi Arabia. This is particularly important given that Saudi Arabia represents a distinct sociocultural and digital environment characterized by high social media penetration, a young and highly connected population, and culturally shaped norms governing communication and self-expression, which may influence how personality traits translate into online behaviors.

However, the relationship between personality traits and online behavior has been extensively investigated in international literature (Roos, 2023; Đurić et al., 2024; Gil de Zúñiga et al., 2017; Christiaan and Murti, 2025), thereby highlighting a significant contextual research gap and raising questions about the generalizability of existing findings to non-Western settings. To address this gap, the present study adopts a design that examines the consistency of the association between personality traits and social media usage across different contexts and measurement approaches. In line with the majority of previous research, the study is grounded in the Big Five model of personality, a well-established and extensively validated framework for understanding core personality dimensions and their relationship with online behavioral patterns.

### Theoretical review

#### The Big Five model

Personality represents a dynamic organization within the individual of those psychophysical systems that determines his characteristic behavior and thought (Allport, 1961). Multiple models of personality have been proposed by several theorists each representing a distinct theoretical perspective. These include the Five-Factor Model, the Myers–Briggs Type Indicator, Eysenck’s three-factor model, and the seven-factor personality model.

The Five Factor Model, commonly referred to as the “Big Five model”, is the most widely recognized and empirically supported theory within the dispositional approach to personality. This framework views personality as encompassing five major dimensions: extraversion, agreeableness, conscientiousness, neuroticism (commonly referred as emotional stability), and openness to experience, which collectively account for the core structure of individual differences in personality (McCrae and Costa, 1997). In short, agreeableness describes the extent to which individuals seek harmonious and cooperative relationships with others. Extraversion denotes a disposition toward sociability, activity, assertiveness, and positive emotionality. Conscientiousness is characterized by self-regulation, goal orientation, planning, and compliance with norms and rules. Neuroticism reflects a predisposition toward negative emotional states, that include anxiety, sadness, and emotional instability. Openness to experience relates creativity, originality, cognitive complexity, and receptiveness to new ideas and experiences (Costa and McCrae, 1988).

#### Big Five model and social media use

Agreeableness and use of social media: Previous researches investigating the relationship between agreeableness and social media use have revealed ambiguous and often contradictory results. Several studies have identified a positive association between agreeableness and social media usage (Gil de Zúñiga et al., 2017; Seidman, 2013). Individuals high in agreeableness are more likely to engage with social media platforms, such as Facebook, to fulfil social belonging needs, gain peer acceptance, and foster interpersonal relationships (Seidman, 2013). Other studies have failed to demonstrate any significant association between agreeableness and social media use (Roos, 2023). Furthermore, some researches indicate that as agreeableness is closely linked to conflict avoidance and interpersonal harmony, highly agreeable individuals may deliberately limit their social media engagement due to its public visibility and the increased likelihood of exposure to disagreement or negative interactions (Barnes et al., 2017). Taken together, these divergent findings provide limited and inconsistent empirical support for a definitive relationship between agreeableness and social media use. Notably, the Big Five personality model offers minimal theoretical guidance for predicting how agreeableness manifests in online behavior. Social media platforms can thus serve as dual-purpose environments, enabling highly agreeable individuals to express supportive and prosocial content, while simultaneously allowing individuals lower in agreeableness to engage in more confrontational or hostile interactions. Accordingly, the following hypotheses are formulated.

*H1a: Agreeableness will be positively related to socialization motives for social media use.*

*H1b: Agreeableness will be positively related to informativeness motives for social media use.*

Extraversion and use of social media: The theoretical understanding of how extraversion influences social media habits has changed over the years. Early research, guided by the social compensation hypothesis, proposed that individuals with lower levels of extraversion would be more drawn to online platforms. This was based on the idea that the digital environment alleviates social anxiety and facilitates communication without face-to-face contact, thus compensating for a lack of real-world social interaction (Hamburger and Ben-Artzi, 2000; Amichai-Hamburger et al., 2002; Goby, 2006). Conversely, the rich-get-richer framework offers a competing perspective, suggesting that socially confident individuals are better positioned to leverage digital platforms. From this viewpoint, these users employ social media primarily to strengthen their existing relationships and extend their social networks further (Kraut et al., 2002).

Contemporary empirical studies consistently validate the rich-get-richer model, finding that extraverts demonstrate significantly higher levels of engagement with social networking platforms (Correa et al., 2010; Ross et al., 2009; Ryan and Xenos, 2011; Roos and Kazemi, 2021; Wang et al., 2012). This trend can likely be explained by the evolution of online platforms, which increasingly prioritize rapid exchanges, immediate feedback, and emotionally charged content—characteristics that resonate with extraverts’ natural preference for high stimulation, social interaction, and positive affect (Tan and Yang, 2014; Roos and Kazemi, 2021; Quintelier and Theocharis, 2013). Consequently, today’s digital spaces appear to disproportionately facilitate engagement among individuals with higher levels of extraversion. Based on this accumulated evidence, the following hypotheses are proposed:

*H2a: Extraversion will be positively related to socialization motives for social media use.*

*H2b: Extraversion will be positively related to entertainment motives for social media use.*

Conscientiousness and use of social media: Studies exploring the link between conscientiousness and social media engagement has yielded inconsistent results. Several studies have indicated a negative relationship between conscientiousness and social media use (Roos, 2023), suggesting that individuals high in conscientiousness may deliberately curtail their engagement with social networking platforms to minimize distraction and procrastination, thus prioritizing task completion and goal attainment (Ryan and Xenos, 2011; Quintelier and Theocharis, 2013). On the other hand, other research have documented a positive association, suggesting that conscientious individuals might leverage social media strategically for professional development, networking, career advancement, and other work-related purposes (Özgüven and Mucan, 2013; Gil de Zúñiga et al., 2017). Furthermore, highly conscientious individuals tend to exhibit a cautious behavioral style, which make them less prone to sharing personal content, such as photos or comments, compared with more impulsive users (Seidman, 2013).

From a theoretical perspective, the Big Five model accommodates both observed patterns (McCrae and Costa, 1997). Social media might serves as an efficient tool for organized and goal-oriented individuals seeking to enhance professional opportunities. Conversely, for those with lower levels of self-regulation and ambition, these platforms may primarily functions as a source of entertainment and relaxation. In light of these conflicting empirical evidence and theoretical reasoning, the direction of the relationship between conscientiousness and social media use remains uncertain. Therefore, the following hypotheses are proposed:

*H3a: Conscientiousness will be positively related to academic motives for social media use.*

*H3b: Conscientiousness will be negatively related to entertainment motives for social media use.*

Neuroticism and use of social media: In the Big Five personality framework, neuroticism is characterized by a dispositional tendency toward emotional instability, anxiety, and heightened stress reactivity (McCrae and Costa, 1997). Accumulating empirical evidence shows that individuals scoring high on neuroticism use social media platforms more frequently than their emotionally stable counterparts (Correa et al., 2010; Gil de Zúñiga et al., 2017). Prior research suggests that such tendency is frequently driven by a desire to manage negative affect, obtain social validation, and compensate for perceived lack in offline social support (Seidman, 2013; Gill et al., 2009). Earlier studies on general internet use yielded inconclusive findings, with some studies reporting avoidance among highly neurotic individuals due to privacy concerns and stress sensitivity associated with certain online tasks (Tuten and Bosnjak, 2001). However, more recent research have clarified that socially and emotionally oriented online activities—particularly social networking, blogging, and leisure-related use—are correlated positively with neuroticism (Correa et al., 2010; Guadagno et al., 2008; Mark and Ganzach, 2014). Social media environments may be especially appealing to highly neurotic individuals because they offer opportunities for identity expression, provide immediate social feedback, and provide passive relaxation content, all of which can aid in anxiety reduction and emotional regulation (Amichai-Hamburger et al., 2002; Amiel and Sargent, 2004; Swickert et al., 2002). In summary, the preponderance of evidence supports a positive association between neuroticism and social media use, particularly when engagement serves affect-regulatory and social-support functions, though the precise strength of this link is likely contingent on the nature of the activity. Based on this, the following hypotheses are advanced:

*H4a: Neuroticism will be positively related to socialization motives for social media use.*

*H4b: Neuroticism will be positively related to entertainment motives for social media use.*

Openness to Experience and Use of Social Media: Empirical evidence suggests a systematic link between openness to experience and greater engagement with digital media. Initial investigations reported that individuals high in openness tend to engage in exploratory online behaviors, such as casual browsing, entertainment-oriented activities, and targeted information searches (Tuten and Bosnjak, 2001). Building on this foundation, later studies demonstrated that open individuals show a greater propensity to embrace and experiment with emerging internet applications, such as blogs (Guadagno et al., 2008), and social networking platforms (Correa et al., 2010). Contemporary research further confirms that openness to experience is positively associated with both overall internet use and diverse online activity (Mark and Ganzach, 2014; Kim and Jeong, 2015). In the context of social media, individuals characterized by high openness demonstrate heightened levels of active participation, including content sharing, interacting with other, and utilizing platforms for social learning and organizing offline activities (Gil de Zúñiga et al., 2017; Correa et al., 2010; Ross et al., 2009; Özgüven and Mucan, 2013; Carpenter et al., 2011). This is theoretically coherent, as the Big Five framework conceptualizes openness as reflecting curiosity, cognitive flexibility, and receptiveness to new experiences, characteristics that may facilitate engagement with continuously evolving digital technologies (McCrae and Costa, 1997; Chamorro-Premuzic, 2012). Taken together, the evidence provide a compelling rationale for expecting a positive association between openness to experience and internet and particularly social media use. However, given that existing empirical findings and theoretical arguments derived from the Big Five framework lend support to competing expectations, the direction of the association remains ambiguous. Based on the previous research briefly outlined above, we proposed the following hypotheses:

*H5a: Openness will be positively related to informativeness motives for social media use.*

*H5b: Openness will be positively related to academic motives for social media use.*

## Materials and methods

### Study design

The present study employed a cross-sectional observational design to collect data during the period from December 2025 to January 2026. Ethical approval for the study protocol was obtained from the Deanship of Scientific Research at King Faisal University, Al-Ahsa, Saudi Arabia (Approval No. KFU-REC-2025-DEC-ETHICS3916). All study procedures were conducted in compliance with the ethical principles and regulatory standards articulated in the Declaration of Helsinki for research involving human participants. Prior to enrollment, participants were provided with detailed written information regarding the study objectives, procedures, potential risks, and anticipated benefits. Participation was voluntary, and informed consent was obtained from all participants before data collection. Measures were implemented to ensure data confidentiality, participant anonymity, and adherence to institutional and international ethical requirements throughout the study period.

### Study sample

The study sample consisted of undergraduate medical students enrolled in the Bachelor of Medicine and Bachelor of Surgery (MBBS) program at King Faisal University. The minimum required sample size was estimated to be 313 participants using Cochran’s modified formula (Cochran, 1977), with finite population correction, based on a total population of 1,687 students, a 95% confidence level, a 5% margin of error, and an assumed population proportion of 0.5. This approach ensures adequate precision and representativeness of population estimates in cross-sectional studies (Cochran, 1977). To account for potential non-response, incomplete questionnaires, and to enhance the statistical power and representativeness of the study, the target sample size was increased to 400 participants. A convenience sampling method was employed, and students were invited through direct contact to participate. Out of 400 students approached, 370 completed the questionnaire in full and were included in the final analysis, yielding a response rate of 92.5%. The final achieved sample size exceeded the minimum required sample, thereby improving the precision of the estimates within the defined margin of error. The socio-demographic characteristics of the study sample are presented in Table 1. Inclusion criteria comprised MBBS students in Year 1 to Year 5 of the program and Saudi medical students. Non-medical students and those who did not provide informed consent were excluded. All participants were provided with detailed information regarding the study objectives, and participation was entirely voluntary.

**Table 1:** Demographic characteristics of participants (*N* = 370).

Variables	Frequency	%
Average time using social media		
<2 h	28	7.6
2–3 h	66	17.8
4–5 h	98	26.5
6–7 h	108	29.2
>7 h	70	18.9
Gender		
Male	234	63.2
Female	136	36.8
Age		
18–20 years	176	47.6
21–23 years	140	37.8
>23 years	54	14.6
Academic year		
First year	103	27.8
Second year	62	16.8
Third year	151	40.8
Fourth year	37	10.0
Fifth year	17	4.6
GPA		
4.5–5	155	41.9
4–4.4	112	30.3
3–3.9	71	19.2
<3	32	8.6
Family status		
Nuclear family	203	54.9
Joint family	167	45.1
Area of residence		
Urban	312	84.3
Rural	58	15.7
Average family income (SAR)		
<5,000	29	7.8
5,001–10,000	67	18.1
10,001–15,000	79	21.4
>15,001	195	52.7
Type of stay		
Living with family	302	81.6
University housing	43	11.6
Sharing apartment	25	6.8
Number of friends on social media		
<100	276	74.6
≥101	94	25.4

### Data collection tools

To achieve the objectives of the present study, data were collected using a structured questionnaire comprising three distinct sections. The first section gathered socio-demographic information, including participants’ gender, academic year, family structure, and family income. Participants were also asked to report their grade point average (GPA) for the previous semester on a standardized 5-point scale, where a score of 1 corresponded to a GPA between 4.5 and 5.0, and a score of 4 corresponded to a GPA below 3.0. This approach allowed for a uniform assessment of academic performance across all respondents. Moreover, we measured total screen time spend on social media. Participants were asked to report the total time they spend on social media each day. Responses were recorded using a 5-point scale where ‘1’ indicated <2 h and ‘5’ represented >7 h of daily social media use. The second section employed the Social Networking Usage Questionnaire (SNUQ) developed by Gupta and Bashir (2018), a 19-item instrument designed to assess patterns of social media use across four domains: academic purposes (7 items), socialization (5 items), entertainment (4 items), and information seeking (3 items). Participants rated each item on a 5-point Likert scale ranging from 1 (“never”) to 5 (“always”), with higher scores indicating greater engagement with social networking sites. Participants were instructed to report their typical social media usage over the past month, consistent with common practice in self-reported behavioral frequency measures. Although the SNUQ was not originally developed as a scale measuring motives, its structure encompasses the key dimensions of students’ motivations for using social media, as supported by recent empirical research (AlFaris et al., 2018; Masalimova et al., 2023). The instrument was initially validated in India and has been effectively applied in Arab contexts (Ibrahim et al., 2024; Hama amin and Mohammad, 2024). Its original validation demonstrated good internal consistency (Cronbach’s *α* = 0.83), confirming its reliability. In the present study, internal consistency of SNUQ demonstrated good reliability (Cronbach’s *α* = 0.84), with subscale coefficients as follows: academic purposes (*α* = 0.79), socialization (*α* = 0.71), entertainment (*α* = 0.66), and information seeking (*α* = 0.62). Given its cross-cultural applicability and relevance to the conceptualization of social media usage motives in this study, the SNUQ was deemed suitable for assessing the patterns of SNS engagement among Saudi medical students. The third section assessed personality traits using the Big Five Inventory–10 (BFI-10) (Rammstedt and John, 2007), which evaluates the five core dimensions of personality: Extraversion (items 1 and 6), Agreeableness (items 2 and 7), Conscientiousness (items 3 and 8), Neuroticism (items 4 and 9), and Openness to Experience (items 5 and 10). Each trait is measured by two items, one positively worded and one negatively worded, rated on a 5-point Likert scale from 1 (“strongly disagree”) to 5 (“strongly agree”). Subscale scores were summed to produce total scores ranging from 10 to 50, with higher scores reflecting greater levels of the respective trait. Roos et al. (2020) reported low inter-item correlations for BFI-10 personality dimensions, ranging from 0.03 to 0.46 across traits. Given the two-item structure of each BFI-10 subscale, internal consistency was evaluated using inter-item correlations rather than Cronbach’s alpha, in line with methodological recommendations. The inter-item correlations for the five personality dimensions were as follows: extraversion (*r* = 0.06), agreeableness (*r* = 0.10), conscientiousness (*r* = 0.13), neuroticism (*r* = 0.04), and openness to experience (*r* = 0.10), indicating low reliability for brief measures.

### Procedure

A pilot study involving 30 students was conducted prior to the main survey to ensure the reliability and validity of the data collection instrument. Data from these students were not included in the final analysis. Participant recruitment was supported by trained medical students. As each academic cohort maintains a designated social media communication group, the respective WhatsApp group administrators were approached to inform students about the study objectives. Following this, email contact details were collected, and students were individually invited via email to confirm their voluntary participation. Data collection commenced only after written informed consent was obtained, at which point participants completed the questionnaire.

### Data analysis

Statistical Package for Social Science software (SPSS, version 30) was used for statistical analysis. Descriptive statistics, including frequencies and percentages, were used to summarize the sociodemographic characteristics prior to conducting inferential analyses. Assumptions for inferential statistics were assessed by examining data normality, linearity, and multicollinearity. Multicollinearity was evaluated using the variance inflation factor (VIF) and tolerance values; all VIF values were below 5.0, and tolerance values exceeded 0.20, indicating the absence of serious multicollinearity. In addition, linearity was assessed before model estimation through scatterplots of standardized residuals against predicted values. All assumptions for inferential statistical analyses were satisfied. The dependent variables were continuous, and independent variables included both continuous and categorical predictors. Hierarchical multiple regression was used to examine the association between Big Five personal factors and motives to use social media after controlled by average daily social media time, gender, age, academic year, family status, area of residence, family income, GPA and number of social media friends. The control variables were entered in Model 1, followed by personality factors (Big Five) in Model 2 to assess their progressive explanatory power. The model fit was assessed using *R*^2^ and changes in *R*^2^ statistical significance of variance explained by the predictor entered at each step was assessed by *F* for change in *R*^2^. Regression coefficients are described as unstandardized coefficients (b), standard error (SE), and standardized beta coefficient (*β*) to explain the effect size. All statistical tests were two-tailed with statistical significance set at <0.05.

## Results

Table 1 provides a detailed overview of the demographic characteristics of the 370 participants. Overall, the sample demonstrated a high level of daily social media engagement, with most participants reporting four or more hours of use per day. The sample was predominantly male and largely comprised younger students aged 18–20 years. A considerable proportion of participants were in their third academic year and reported high academic performance. Additionally, most participants resided in urban areas, lived with their families, and reported relatively higher family income levels.

To test the proposed hypotheses, a series of regression analyses were performed to assess the predictive role of Big Five personality traits on social media use motives.

### Agreeableness as a predictor of social media use (H1a and H1b)

Table 2 presents hierarchical regression analyses examining the predictive role of agreeableness on socialization (H1a) and informational (H1b) motives for social media use, after controlling for average daily social media time, gender, age, academic year, family status, area of residence, family income, GPA and number of social media friends.

**Table 2:** Hierarchical regression models assessing agreeableness in relation to socialization and informativeness motives for social media use, with sociodemographic and usage controls.

Variables	Socialization motive						Informativeness motive					
	Model 1			Model 2			Model 1			Model 2		
	*b*	*SE*	*β*	*b*	*SE*	*β*	*b*	*SE*	*β*	*b*	*SE*	*β*
Average time	0.49	0.14	0.18**	0.49	0.14	0.17**	0.29	0.09	0.15**	0.29	0.09	0.15**
Gender	1.52	0.43	0.19**	1.52	0.43	0.19**	0.95	0.29	0.18**	0.95	0.29	0.18**
Age	−0.68	0.54	−0.13	−0.62	0.54	−0.12	−1.00	0.37	−0.28**	−0.99	0.37	−0.27**
Academic year	−0.29	0.35	−0.09	−0.35	0.36	−0.10	0.41	0.24	0.18	0.40	0.24	0.17
Family status	0.29	0.38	0.04	0.36	0.38	0.05	0.51	0.26	0.09*	0.52	0.26	0.10*
Area of residence	0.21	0.53	0.02	0.20	0.53	0.02	−0.43	0.36	−0.06	−0.43	0.36	−0.06
Monthly income	−0.23	0.20	−0.06	−0.25	0.20	−0.07	−0.36	0.14	−0.14**	−0.36	0.14	−0.014**
Type of stay	−0.20	0.33	0.03	−0.21	0.33	−0.03	0.31	0.23	0.07	0.31	0.23	0.07
GPA	0.11	0.20	0.03	0.15	0.20	0.04	0.08	0.14	0.03	0.09	0.14	0.04
Social media friends	1.61	0.44	0.18**	1.63	0.44	0.19**	0.94	0.30	0.16**	0.94	0.29	0.16**
Agreeableness				0.17	0.11	0.08				0.04	0.08	0.03
*R* ^2^	0.14			0.15			0.13			0.14		
*R*^2^ change	0.14			0.01			0.13			0.01		
*F* change	5.94**			2.29			5.21**			0.23		

**p < 0.01; *p < 0.05.

For socialization motives, Model 1, including only control variables was significant (*R*^2^ = 0.14, FΔ = 5.94, *p* < 0.01). Average time spent on social media (*β* = 0.18, *p* < 0.01) gender (*β* = 0.19, *p* < 0.01) and social media friends (*β* = 0.18, *p* < 0.01) positively predicted socialization motives, whereas age, academic year, family status, monthly income, type of stay, GPA and area of residence were not significant. After adding agreeableness in Model 2, it did not significantly predict socialization motives (*β* = 0.08, *p* > 0.05), with minimal change in explained variance (Δ*R*^2^ = 0.01), indicating that H1a was not supported.

For informativeness motives, Model 1 explained 13% of the variance (*R*^2^ = 0.13, *F*Δ = 5.21, *p* < 0.01). Average time (*β* = 0.15, *p* < 0.01), gender (β = 0.18, *p* < 0.01) family status (*β* = 0.09, *p* < 0.05), and social media friends (*β* = 0.16, *p* < 0.01) were positive predictors, and age (*β* = −0.28, *p* < 0.01) and monthly income (*β* = −0.14, *p* < 0.01) were negatively associated. Adding agreeableness in Model 2 did not significantly increase explained variance (*β* = 0.03, *p* > 0.05, Δ*R*^2^ = 0.01), failing to support H1b. These results suggest that sociodemographic and usage variables, rather than agreeableness, account for variation in these motives.

### Extraversion as a predictor of social media use motives (H2a and H2b)

Table 3 summarizes the hierarchical regression analyses for extraversion. For socialization motives, Model 1 (controls only) explained 14% of variance (*R*^2^ = 0.14, FΔ = 6.00, *p* < 0.01), with average time (*β* = 0.18, *p* < 0.01), gender (β = 0.19, *p* < 0.01) and social media friends (*β* = 0.18, *p* < 0.01) as significant positive predictors. Extraversion added in Model 2 significantly predicted socialization motives (*β* = 0.17, *p* < 0.01), increasing explained variance by 3% (Δ*R*^2^ = 0.03, FΔ = 11.34, *p* < 0.01), supporting H2a.

**Table 3:** Hierarchical regression models assessing extraversion in relation to socialization and entertainment motives for social media use with sociodemographic and usage controls.

Variables	Socialization motive						Entertainment motive					
	Model 1			Model 2			Model 1			Model 2		
	*b*	*SE*	*β*	*b*	*SE*	*β*	*b*	*SE*	*β*	*b*	*SE*	β
Average time	0.50	0.14	0.18**	0.49	0.14	0.17**	0.59	0.12	0.24**	0.55	0.12	0.23**
Gender	1.55	0.43	0.19**	1.52	0.43	0.19**	1.27	0.36	0.19**	1.25	0.35	0.19**
Age	−0.67	0.54	−0.13	−0.46	0.54	−0.12	−0.34	0.45	−0.08**	−0.18	0.45	−0.04
Academic year	−0.30	0.35	0.09	−0.39	0.36	−0.10	0.03	0.30	0.01	−0.04	0.29	0.02
Family status	0.29	0.38	0.04	0.40	0.38	0.05	0.07	0.32	0.01	0.16	0.31	0.03
Area of residence	0.21	0.53	0.02	0.40	0.53	0.02	0.13	0.44	0.02	0.28	0.44	0.03
Monthly income	−0.23	0.20	−0.06	−0.27	0.20	−0.07	0.34	0.17	0.11*	0.31	0.16	0.09
Type of stay	−0.20	0.33	−0.03	−0.26	0.33	−0.03	0.09	0.28	0.02	0.04	0.27	0.01
GPA	0.11	0.20	0.03	0.13	0.20	0.04	0.24	0.17	0.08	0.25	0.16	0.08
Social media friends	1.61	0.44	0.18**	1.57	0.44	0.19**	0.98	0.36	0.14**	0.95	0.36	0.13**
Extraversion				0.38	0.11	0.17**				0.30	0.09	0.16**
*R* ^2^	0.14			0.17			0.14			0.16		
*R*^2^ Change	0.14			0.03			0.14			0.02		
*F* Change	6.00**			11.34**			5.69**			9.95**		

**p < 0.01; *p < 0.05.

For entertainment motives, Model 1 explained 14% of variance (*R*^2^ = 0.14, *F*Δ = 5.69, *p* < 0.01). Average time (*β* = 0.24, *p* < 0.01), gender (*β* = 0.19, *p* < 0.01) monthly income (*β* = 0.11, *p* < 0.05) and social media friends (*β* = 0.14, *p* < 0.01) were positive predictors, and age negatively predicted entertainment motives (*β* = −0.08, *p* < 0.01). Extraversion in Model 2 positively predicted entertainment motives (*β* = 0.16, *p* < 0.01), with a 2% increase in explained variance (Δ*R*^2^ = 0.02, *F*Δ = 9.95, *p* < 0.01), supporting H2b. Overall, extraversion consistently predicted both socialization and entertainment motives beyond the effect of control variables.

### Conscientiousness as a predictor of social media use motives (H3a and H3b)

Table 4 presents the hierarchical regression results for conscientiousness. For academic motives, Model 1, average time spent on social media (*β* = 0.13, *p* < 0.01) and gender (*β* = 0.29, *p* < 0.01) positively predicted socialization motives, whereas age (*β* = −0.20, *p* < 0.01) and GPA (*β* = −0.18, *p* < 0.01) are negatively associated. The model explained 13% of variance (*R*^2^ = 0.13, FΔ = 5.52, *p* < 0.01). Adding conscientiousness in Model 2 did not predict academic motives (*β* = 0.04, *p* > 0.05), with a negligible increase in variance (Δ*R*^2^ = 0.01), failing to support H3a.

**Table 4:** Hierarchical regression models of conscientiousness predicting academic and entertainment motives for social media use with sociodemographic and usage controls.

Variables	Academic motives						Entertainment motive					
	Model 1			Model 2			Model 1			Model 2		
	*b*	*SE*	*β*	*b*	*SE*	*β*	*b*	*SE*	*β*	*b*	*SE*	*β*
Average time	0.54	0.21	0.13**	0.56	0.21	0.14**	0.59	0.12	0.24**	0.51	0.12	0.22**
Gender	3.23	0.62	0.29**	3.31	0.62	0.29**	1.27	0.36	0.19**	1.30	0.35	0.20**
Age	−1.56	0.79	−0.20**	−1.49	0.79	−0.19	−0.34	0.45	−0.08**	−0.51	0.45	−0.12
Academic year	0.81	0.51	0.17	0.78	0.52	0.16	0.03	0.30	0.01	0.10	0.29	0.04
Family status	−0.14	0.55	−0.01	−0.17	0.55	−0.02	0.07	0.32	0.01	0.12	0.31	0.02
Area of residence	−0.41	0.77	−0.03	−0.46	0.77	−0.03	0.13	0.44	0.02	0.25	0.44	0.03
Monthly income	−0.21	0.29	−0.04	−0.18	0.29	−0.03	0.34	0.17	0.11*	0.27	0.17	0.09
Type of stay	−0.14	0.48	−0.02	−0.14	0.48	−0.02	0.09	0.28	0.02	0.09	0.27	0.02
GPA	−0.99	0.29	−0.18**	−1.00	0.29	−0.18**	0.24	0.17	0.08	0.26	0.16	0.08
Social media friends	0.52	0.63	0.04	0.49	0.63	0.04	0.98	0.36	0.14*	1.06	0.36	0.15**
Conscientiousness				−0.15	0.19	−0.04				0.34	0.11	0.16**
*R* ^2^	0.13			0.14			0.14			0.16		
*R*^2^ change	0.13			0.01			0.14			0.02		
*F* change	5.52**			0.57			5.69**			9.68**		

**p < 0.01; *p < 0.05.

For entertainment motives, Model 1 showed positive associations for average time (*β* = 0.24, *p* < 0.01), gender (*β* = 0.19, *p* < 0.01), monthly income (*β* = 0.11, *p* < 0.05), and social media friends (*β* = 0.14, *p* < 0.01), and a negative effect of age (*β* = −0.08, *p* < 0.01), explaining 14% of variance. Conscientiousness in Model 2 positively predicted entertainment motives (*β* = 0.16, *p* < 0.01), increasing explained variance by 2% (Δ*R*^2^ = 0.02, *F*Δ = 9.68, *p* < 0.01), contrary to expectations, indicating that more conscientious individuals may also engage in entertainment-oriented social media use.

### Neuroticism as a predictor of social media use motives (H4a and H4b)

Table 5 summarizes the effects of neuroticism. For socialization motives, Model 1 indicated significant positive predictors for average time (*β* = 0.18, *p* < 0.01), gender (*β* = 0.20, *p* < 0.01), and social media friends (*β* = 0.18, *p* < 0.01), explaining 14% of variance. Neuroticism in Model 2 significantly predicted socialization motives (*β* = 0.15, *p* < 0.01), increasing the variance explained to 16% (Δ*R*^2^ = 0.16, *F*Δ = 8.70, *p* < 0.01), supporting H4a.

**Table 5:** Hierarchical regression models of neuroticism predicting socialization and entertainment motives for social media use with sociodemographic and usage controls.

Variables	Socialization motives						Entertainment motive					
	Model 1			Model 2			Model 1			Model 2		
	*b*	*SE*	*β*	*b*	*SE*	*β*	*b*	*SE*	*β*	*b*	*SE*	*β*
Average time	0.50	0.14	0.18**	0.47	0.14	0.17**	0.59	0.12	0.24**	0.53	0.12	0.29**
Gender	1.55	0.43	0.20**	1.58	0.42	0.20**	1.27	0.36	0.19**	1.30	0.35	0.20**
Age	−0.67	0.54	−0.13	−0.74	0.58	−0.14	−0.34	0.45	−0.08**	−0.40	0.45	−0.09
Academic year	−0.30	0.35	−0.09	−0.21	0.36	−0.06	0.03	0.30	0.01	0.11	0.29	0.04
Family status	0.29	0.38	0.04	0.26	0.38	0.03	0.07	0.32	0.01	0.04	0.31	0.01
Area of residence	0.21	0.53	0.02	0.30	0.53	0.03	0.13	0.44	0.02	0.20	0.44	0.02
Monthly income	−0.23	0.20	−0.06	−0.29	0.20	−0.08	0.34	0.17	0.11*	0.29	0.17	0.09
Type of stay	−0.20	0.33	−0.03	−0.18	0.33	−0.03	0.09	0.28	0.02	0.11	0.27	0.02
GPA	0.11	0.20	0.03	0.08	0.20	0.02	0.24	0.17	0.08	0.22	0.16	0.07
Social media friends	1.60	0.44	0.18**	1.73	0.43	0.20**	0.98	0.36	0.14*	1.09	0.36	0.15**
Neuroticism				0.39	0.13	0.15**				0.34	0.11	0.16**
*R* ^2^	0.14			0.16			0.14			0.16		
*R*^2^ change	0.14			0.02			0.14			0.02		
*F* change	6.00**			8.70**			5.69**			10.13**		

**p < 0.01; *p < 0.05.

For entertainment motives, Model 1 showed positive effects for average time (*β* = 0.24, *p* < 0.01), gender (*β* = 0.19, *p* < 0.01), monthly income (*β* = 0.11, *p* < 0.05) and social media friends (*β* = 0.14, *p* < 0.01), negative for age (*β* = −0.08, *p* < 0.01), explaining 14% of variance. Neuroticism positively predicted entertainment motives in Model 2 (*β* = 0.16, *p* < 0.01), with a 2% increase in variance (Δ*R*^2^ = 0.02, FΔ = 10.13, *p* < 0.01), supporting H4b. These findings indicate that individuals higher in neuroticism are more likely to use social media for both social and entertainment purposes.

### Openness to experience as a predictor of social media use motives (H5a and H5b)

Table 6 reports hierarchical regression results for openness to experience. For informational motives, Model 1 showed positive effects of average time (*β* = 0.15, *p* < 0.01), gender (*β* = 0.18, *p* < 0.01), family status (*β* = 0.09, *p* < 0.05), and social media friends (*β* = 0.16, *p* < 0.01), and a negative effect of age (*β* = −0.28, *p* < 0.01) and monthly income (*β* = −0.14, *p* < 0.01), explaining 13% of variance. Adding openness in Model 2 positively predicted informational motives (*β* = 0.15, *p* < 0.01), increasing explained variance to 15% (Δ*R*^2^ = 0.15, *F*Δ = 9.90, *p* < 0.01), supporting H5a.

**Table 6:** Hierarchical regression models of openness to experience predicting informational and academic motives for social media use, controlling for sociodemographic and usage variables.

Variables	Informational motives						Academic motives					
	Model 1			Model 2			Model 1			Model 2		
	*b*	*SE*	*β*	*b*	*SE*	*β*	*b*	*SE*	*β*	*b*	*SE*	*β*
Average time	0.29	0.09	0.15**	0.30	0.09	0.16**	0.54	0.21	0.13**	0.54	0.21	0.13**
Gender	0.94	0.29	0.18**	0.94	0.29	0.18**	3.33	0.62	0.29**	3.33	0.62	0.29**
Age	−1.00	0.37	−0.28**	−0.99	0.36	−0.28**	−1.56	0.79	−0.20*	−1.56	0.79	−0.20*
Academic year	0.41	0.24	0.18	0.39	0.24	0.17	0.81	0.51	0.17	0.81	0.54	0.17
Family status	0.50	0.26	0.09*	0.50	0.26	0.09*	−0.14	0.55	−0.01	−0.15	0.55	−0.01
Area of residence	−0.43	0.36	−0.06	−0.50	0.36	−0.07	−0.41	0.77	−0.03	−0.44	0.77	−0.03
Monthly income	−0.36	0.14	−0.14**	−0.42	0.14	−0.16**	−0.21	0.29	−0.04	−0.24	0.29	−0.04
Type of stay	0.31	0.23	0.07	0.33	0.22	0.07	−0.14	0.48	−0.01	−0.13	0.48	−0.01
GPA	0.08	0.14	0.03	0.08	0.13	0.03	−0.99	0.29	−0.18**	−0.99	0.29	−0.18**
Social media friends	0.94	0.30	0.16**	1.02	0.29	0.17**	0.52	0.63	0.04	0.56	0.64	0.04
Openness to experience				0.24	0.07	0.15**				0.11	0.16	0.03
*R* ^2^	0.13			0.15			0.13			0.14		
*R*^2^ change	0.13			0.02			0.13			0.01		
*F* change	5.21**			9.90**			5.51**			0.42		

**p < 0.01; *p < 0.05.

For academic motives, Model 1 indicate positive effect of average time (*β* = 0.13, *p* < 0.01) and gender (*β* = 0.29, *p* < 0.01), and a negative effect of age (*β* = −0.20, *p* < 0.05), and GPA (*β* = −0.18, *p* < 0.01), with variance explained of 13%. Adding openness in Model 2 did not significantly predict academic motives (*β* = 0.03, *p* > 0.05), ΔR^2^ = 0.01, failing to support H5b. Thus, openness relates selectively to exploratory, informational engagement rather than academically oriented social media use.

### Collective contribution of big five personality traits to social media use motives

Table 7 presents the hierarchical regression examining the combined effect of all Big Five traits on overall social media motives. Model 1 (controls only) explained 17% of variance, with average time (*β* = 0.23, *p* < 0.01), gender (*β* = 0.30, *p* < 0.01), age (*β* = −0.22, *p* < 0.05) and social media friends (*β* = 0.15, *p* < 0.01) as significant predictors. Adding the Big Five traits collectively in Model 2 significantly improved the model, explaining an additional 3% of variance (Δ*R*^2^ = 0.03, *F*Δ = 14.39, *p* < 0.01), with a total *R*^2^ = 0.20. Overall, personality traits together were significant predictors (*β* = 0.18, *p* < 0.01), highlighting their unique contribution to explaining social media motivation beyond demographic and usage factors.

**Table 7:** Hierarchical regression models of Big Five personality traits predicting social media use motives, controlling for demographic and usage variables.

Variables	Social media motives					
	Model 1			Model 2		
	*b*	*SE*	*β*	*b*	*SE*	*β*
Average time	0.47	0.10	0.23**	0.45	0.10	0.21**
Gender	1.77	0.31	0.30**	1.78	0.31	0.30**
Age	−0.89	0.40	−0.22*	−0.87	0.39	−0.22*
Academic year	0.24	0.26	0.09	0.21	0.26	0.08
Family status	0.18	0.28	0.03	0.26	0.27	0.05
Area of residence	−0.12	0.39	−0.02	−0.04	0.38	−0.01
Monthly income	−0.11	0.15	−0.04	−0.21	0.15	−0.07
Type of stay	0.02	0.24	0.01	0.01	0.24	0.01
GPA	−0.14	0.15	−0.05	−0.10	0.14	−0.04
Social media friends	1.01	0.32	0.15**	1.12	0.31	0.17**
Big Five traits				0.57	0.15	0.18**
*R*2	0.17			0.20		
*R*^2^ change	0.17			0.03		
*F* change	7.43**			14.39**		

**p < 0.01; *p < 0.05.

## Discussion

To the best of our knowledge, this study represents the first investigation in Saudi Arabia to comprehensively explore how Big Five personality traits predict distinct motives for social media use among medical students, while accounting for sociodemographic characteristics and patterns of social media engagement. By examining multiple motivational domains concurrently, the findings provide a deeper and more nuanced understanding of the psychological mechanisms underlying social media use, demonstrating that personality traits are differentially associated with socialization, informational, entertainment, and academic motives. Importantly, these associations persist beyond the influence of time spent online, gender, and social network size, underscoring the unique contribution of stable personality characteristics in shaping motivational orientations toward social media. Collectively, the results highlight the relevance of individual differences in explaining variability in social media use among medical students and offer theoretical support for personality-based models of media use within academic and professional training contexts.

### Agreeableness and social media motives

Contrary to expectations, agreeableness was not significantly associated with either socialization or informational motives for social media use. Although individuals high in agreeableness are typically described as cooperative, empathetic, and interpersonally oriented (Costa and McCrae, 1992), these characteristics did not translate into stronger motivation to engage with social media for social interaction or information seeking in the present sample. This finding is consistent with previous research reporting non-significant associations between agreeableness and various dimensions of social media use (Roos, 2023). One possible explanation is that agreeableness may be more reflective of the quality of interpersonal relationships rather than the frequency or motivational drivers of engagement on digital platforms. In the context of medical education, students may prioritize face-to-face communication, formal academic collaboration, or structured professional interactions, thereby reducing the relevance of agreeableness for online socialization or information-seeking behaviors. Moreover, the highly demanding and competitive nature of medical training may attenuate the influence of interpersonal personality traits on discretionary social media motives. Notably, usage-related variables, particularly time spent on social media and the size of one’s social network, emerged as consistent predictors of socialization and informational motives. This suggests that structural and behavioral aspects of social media engagement may play a more prominent role than dispositional traits such as agreeableness in shaping these motivations among medical students. However, this interpretation should be considered in light of an important contextual limitation. The present study did not collect data on which specific social networking platforms participants primarily used (e.g., messaging-based applications versus content-sharing platforms). Since different platforms are characterized by distinct affordances that shape communication patterns and interaction styles, the absence of such data limits the precision with which behavioral interpretations—such as preferences for face-to-face communication, formal academic collaboration, or structured professional interaction—can be made. Therefore, these interpretations remain tentative and should be refined in future research incorporating platform-specific analyses.

### Extraversion as a key determinant of socialization and entertainment motives

Extraversion emerged as one of the most influential personality traits associated with socialization and entertainment-related motives for social media use, providing strong support for hypotheses H2a and H2b. Students with higher levels of extraversion demonstrated a greater tendency to engage with social media platforms for interpersonal interaction and leisure-oriented activities. The present findings both confirm and expand upon previous research (Roos, 2023; Bowden-Green et al., 2020; Tarar and Baig, 2024; Lin et al., 2024), indicating that extraverted individuals are more active, expressive, and motivated participants in social networking environments. For these individuals, social media appears to function as a digital extension of offline social life, facilitating frequent interaction, entertainment, and social affirmation. Within the highly demanding environment of medical training, marked by heavy academic workloads and constrained free time, social media may provide extraverted students with a convenient and accessible means of satisfying their social and recreational needs. Importantly, the association between extraversion and social media motives persisted independently of time spent online, indicating that these motivations are not simply driven by greater exposure or usage frequency. Rather, the findings suggest that extraversion reflects underlying dispositional orientations that shape how and why individuals engage with digital platforms. Taken together, these results underscore the central role of extraversion in explaining socially and entertainment-driven patterns of social media use.

### Conscientiousness and the unexpected pattern of entertainment use

The findings related to conscientiousness partially contradicted theoretical expectations. Conscientiousness was not significantly associated with academic motives for social media use, leading to the rejection of H3a. This suggests that conscientious students do not necessarily rely on social media for academic purposes, possibly preferring more structured and formal learning resources. Given the rigor of medical training, academically oriented students may view social media as less reliable or less efficient for academic tasks. At the same time, researchers have speculated that conscientiousness may act as a protective factor against the development of problematic social media use (Andreassen et al., 2013), further supporting the notion that conscientious individuals engage with digital platforms in a selective and goal-directed manner.

More unexpectedly, conscientiousness was positively associated with entertainment motives, contradicting H3b. This finding challenges the prevailing assumption that conscientious individuals tend to avoid entertainment-oriented digital activities in favor of productivity-focused behaviors. Prior research has shown that social media use can be positively associated with conscientiousness, with evidence suggesting that highly conscientious individuals often utilize social media for professional networking and work-related purposes (Gil de Zúñiga et al., 2017; Özgüven and Mucan, 2013). Within this broader pattern of goal-directed and regulated media use, it is plausible that conscientious students also engage with entertainment content in a deliberate and controlled manner, rather than as a form of procrastination or distraction.

In high-pressure academic environments such as medical education—characterized by sustained cognitive demands and emotional strain—even highly conscientious individuals may seek controlled forms of entertainment to restore attentional resources and psychological balance. From this perspective, conscientiousness may not reduce engagement with entertainment-related content per se, but instead influence how such content is integrated into daily routines. Accordingly, conscientious individuals may be more likely to regulate the timing, duration, and purpose of entertainment-oriented social media use, ensuring that it serves a restorative function rather than interfering with academic responsibilities. These findings suggest that conscientiousness shapes the mode and intent of entertainment use, rather than its overall occurrence.

### Neuroticism and emotion-driven social media engagement

Neuroticism demonstrated a significant positive association with both socialization and entertainment motives, thereby providing strong support for H4a and H4b. Students with higher levels of neuroticism reported greater motivation to use social media for maintaining interpersonal connections and for entertainment purposes, even after controlling for demographic variables and usage intensity. This finding is consistent with prior research indicating a positive relationship between neuroticism and increased social media engagement (Correa et al., 2010; Gil de Zúñiga et al., 2017; Seidman, 2013; Gill et al., 2009; Marengo et al., 2020).

Social media platforms may offer emotionally reactive individuals a perceived sense of connection, distraction, or reassurance, particularly within high-pressure contexts such as medical education. Entertainment-oriented content may function as an emotion-focused coping strategy to regulate distress (Wolfers and Schneider, 2021), while using social media may provide validation and temporary relief from stress (Wu and Pei, 2022). The concurrent association with both social and entertainment motives suggests that neuroticism exerts a broad influence on emotionally driven engagement patterns rather than being confined to a single motivational domain. Importantly, these relationships remained significant after accounting for time spent on social media, indicating that neuroticism shapes the underlying reasons for engagement rather than merely the frequency of use.

### Openness to experience and selective information-seeking

Openness to experience demonstrated a domain-specific pattern of association, supporting H5a but not H5b. Individuals higher in openness were significantly more likely to engage with social media for informational purposes, whereas no significant relationship emerged between openness and academically oriented motives. These results align with prior research indicating that openness is positively associated with greater engagement and intensity of social media use (Ross et al., 2009; Roos and Kazemi, 2021; Correa et al., 2010; Gil de Zúñiga et al., 2017; Özgüven and Mucan, 2013). Theoretically, openness to experience encompasses intellectual curiosity, cognitive flexibility, and a preference for novelty and innovation, in contrast to lower levels of openness, which are characterized by adherence to routine, tradition, and conventional modes of behavior (John and Srivastava, 1999).

Given that social networking platforms constitute dynamic and continuously evolving digital environments, individuals high in openness are naturally inclined to explore these spaces as sources of new information, perspectives, and experiences (Manner and Lane, 2017). Social media platforms provide rapid access to diverse viewpoints, news, and informal knowledge, which may be particularly appealing to highly open individuals. However, academic motives especially within demanding fields such as medical education often require depth, credibility, and formally validated sources, which social media does not consistently offer. Consequently, openness appears to facilitate exploratory and informal information-seeking rather than structured academic engagement. This differentiated pattern highlights the importance of distinguishing between informational and academic motives, as they represent qualitatively distinct modes of knowledge engagement within digital environments.

### Collective influence of Big Five traits

When considered collectively, the Big Five personality traits significantly predicted overall social media motives, beyond demographic variables and usage patterns. Although the additional variance explained was modest, the effect was statistically meaningful and theoretically important. This finding supports integrative models of digital behavior that emphasize personality as a foundational psychological determinant shaping engagement motivations (Özgüven and Mucan, 2013; Roos and Kazemi, 2021; Drążkowski et al., 2022; Roos, 2023; Alphenaar et al., 2025).

The cumulative contribution of personality highlights that motivations for social media use are not driven by isolated traits operating independently, but rather emerge from the combined influence of multiple dispositional tendencies. Each dimension of the Big Five contributes distinct emotional, cognitive, and behavioral orientations such as sociability (extraversion), emotional sensitivity (neuroticism), self-regulation (conscientiousness), curiosity (openness), and interpersonal orientation (agreeableness), which together shape how individuals interpret, engage with, and derive gratification from digital platforms. In multifaceted behavioral contexts such as social media use, where motivations are influenced by platform affordances, academic demands, and social environments, small but consistent personality effects are theoretically expected and empirically meaningful. Moreover, personality traits tend to exert their influence in an additive and interactive manner rather than through singular dominant effects. However, the present results suggest a more nuanced pattern, as certain individual traits (notably openness to experience) accounted for relatively greater variance in specific motivational domains than the combined Big Five model in predicting overall motives. This indicates that while additive and interactive effects are theoretically plausible, in practice some personality dimensions may exert more domain-specific explanatory power than the aggregated model. While individual traits selectively predicted specific motivational domains in the present study, their collective impact suggests a broader dispositional framework underlying engagement behavior. This aligns with contemporary personality theories emphasizing that complex behaviors are best explained by configurations of traits rather than isolated predictors. Particularly within medical education, where students face intense cognitive workloads, emotional pressures, and limited discretionary time—personality-driven motivational tendencies may subtly but consistently guide how social media is used for connection, information, emotional regulation, and leisure.

Importantly, the persistence of personality effects after controlling for time spent on social media and structural usage variables underscores that these traits influence the qualitative nature of engagement rather than merely its quantity. In other words, personality shapes why students use social media, not simply how frequently they do so. Collectively, these findings reinforce the value of personality-based frameworks in understanding digital behavior and suggest that individual differences play a central role in explaining variability in social media motives among medical students. By situating dispositional traits within the shared context of medical education characterized by inherently demanding academic training, the present study contributes to a more comprehensive psychological understanding of social media use within professional training environments.

This study has several limitations that should be considered when interpreting the findings. First, the cross-sectional design limits causal inference regarding the relationships between Big Five personality traits and social media use motives. Although personality traits are relatively stable over time, longitudinal studies are necessary to examine changes in motivational patterns and potential bidirectional effects between personality and digital engagement behaviors. Second, the reliance on self-reported measures may introduce response and recall biases, particularly in estimating time spent on social media and motivational drivers. While validated instruments were used, future research could enhance measurement precision by incorporating objective usage data or mixed-method approaches, such as digital trace data extracted from smartphone screen-time logs, platform analytics, or experimental behavioral paradigms like the Social Networking Site Behavior Task (Shaw et al., 2022; Pennington et al., 2025), which allow for more ecologically valid assessment of real-time social media engagement. Third, the sample was drawn from a single academic institution using non-probability convenience sampling, which restricts the generalizability of the results. Institutional culture, access to digital resources, and educational practices vary across universities and regions, potentially influencing social media engagement patterns. Consequently, the findings reflect the participating students rather than all medical students in Saudi Arabia or internationally. Future multi-center studies with more diverse and representative samples are recommended to strengthen external validity. Fourth, the study did not collect platform-specific data regarding social media use (e.g., WhatsApp, Instagram, Twitter). Given that different platforms serve distinct social, informational, and entertainment functions, the absence of such differentiation limits the ability to capture qualitative variations in user behavior. Future research should incorporate platform-specific measures to better understand how different forms of social media engagement relate to personality traits and usage motives. Finally, although key demographic and usage-related variables were controlled for, other relevant contextual factors such as academic stress, psychological well-being, platform-specific characteristics, and content types were not assessed. These variables may interact with personality traits in shaping social media motives and should be incorporated into future research to provide a more comprehensive understanding of digital behavior in medical education.

## Conclusion

This study provides novel empirical evidence from Saudi Arabia on the role of Big Five personality traits in shaping distinct motivational patterns of social media use among medical students. By moving beyond simplistic measures of usage frequency and examining multiple motivational domains concurrently, the findings offer a more nuanced psychological understanding of how and why students engage with digital platforms. Personality traits were differentially associated with socialization, informational, entertainment, and academic motives, underscoring the importance of individual differences in explaining variability in social media behavior within academically demanding environments. Extraversion emerged as a key determinant of socially and entertainment-driven engagement, while neuroticism was linked to emotionally motivated use for connection and coping. Openness to experience selectively promoted exploratory and informational engagement, whereas conscientiousness shaped entertainment use in a controlled and restorative manner rather than academic reliance. In contrast, agreeableness did not significantly influence motivational patterns, suggesting that structural and contextual factors may outweigh interpersonal traits in guiding social media engagement among medical students. Importantly, the collective influence of the Big Five traits remained significant beyond demographic and usage-related factors, highlighting personality as a foundational psychological framework underlying digital engagement motivations. These findings lend strong support to personality-based models of media use and emphasize that social media behavior is driven not merely by access or exposure, but by enduring dispositional tendencies interacting with academic and social contexts.

From a practical perspective, the results have important implications for digital well-being initiatives and educational policy in medical training. Understanding that students engage with social media for diverse psychological reasons can inform targeted interventions aimed at promoting healthy, balanced, and purposeful digital engagement. Educational institutions may benefit from integrating structured, credible digital learning resources while also supporting students’ emotional regulation and social needs through appropriate support systems. Overall, this study advances theoretical knowledge of digital behavior in higher education and provides a culturally relevant foundation for future research. By integrating personality psychology with motivational models of media use, the findings contribute to a more comprehensive framework for understanding social media engagement among medical students and highlight the importance of individualized approaches in digital education and student wellbeing.

## Data Availability

The original contributions presented in the study are included in the article/supplementary material, further inquiries can be directed to the corresponding author/s.

## References

[ref1] Ahmed Y. A. Ahmad M. N. Ahmad N. Zakaria N. H. (2019). Social media for knowledge-sharing: a systematic literature review. Telemat. Inform. 37, 72–112. doi: 10.1016/j.tele.2018.01.015

[ref2] AlFaris E. Irfan F. Ponnamperuma G. Jamal A. Van der Vleuten C. Al Maflehi N. . (2018). The pattern of social media use and its association with academic performance among medical students. Med. Teach. 40, S77–S82. doi: 10.1080/0142159X.2018.1465536, 29732945

[ref3] Alfaya M. A. Abdullah N. S. Alshahrani N. Z. Alqahtani A. A. A. Algethami M. R. Al Qahtani A. S. Y. . (2023). Prevalence and determinants of social media addiction among medical students in a selected university in Saudi Arabia: a cross-sectional study. Healthcare 11:1370. doi: 10.3390/healthcare11101370, 37239655 PMC10217812

[ref4] AlHamad N. S. AlAmri K. (2021). The association between social media use and depressive symptoms among adults in Riyadh, Saudi Arabia. J. Fam. Med. Prim. Care 10, 3336–3342. doi: 10.4103/jfmpc.jfmpc_697_21, 34760754 PMC8565162

[ref5] Allport G. W. (1961). Pattern and Growth in Personality. Fort Worth, TX: Harcourt College Publisher.

[ref6] Alphenaar L. E. Shiner R. L. Arana C. C. Prinzie P. (2025). Social media and subjective well-being: the moderating role of personality traits. J. Happiness Stud. 26:61. doi: 10.1007/s10902-025-00898-0

[ref7] Alrasheed M. Alrasheed S. Alqahtani A. S. (2022). Impact of social media exposure on risk perceptions, mental health outcomes, and preventive behaviors during the COVID-19 pandemic in Saudi Arabia. Saudi J. Health Syst. Res. 2, 107–113. doi: 10.1159/000525209

[ref8] Amichai-Hamburger Y. (2007). “Personality, individual differences and internet use,” in The Oxford Handbook of Internet Psychology, eds. Johnson A. McKenna K. Postmes T. Reips U. (Oxford: Oxford University Press), 187–204.

[ref9] Amichai-Hamburger Y. Wainapel G. Fox S. (2002). “On the internet no one knows I’m an introvert”: extraversion, neuroticism, and internet interaction. Cyberpsychol. Behav. 5, 125–128. doi: 10.1089/109493102753770507, 12025878

[ref10] Amiel T. Sargent S. L. (2004). Individual differences in internet usage motives. Comput. Hum. Behav. 20, 711–726. doi: 10.1016/j.chb.2004.09.002

[ref11] Andreassen C. S. Griffiths M. D. Gjertsen S. R. Krossbakken E. Kvam S. Pallesen S. (2013). The relationships between behavioral addictions and the five-factor model of personality. J. Behav. Addict. 2, 90–99. doi: 10.1556/JBA.2.2013.003, 26165928

[ref12] Appel H. Marker C. Gnambs T. (2020). Are social media ruining our lives? A review of meta-analytic evidence. Rev. Gen. Psychol. 24, 60–74. doi: 10.1177/1089268019880891

[ref13] Barnes R. Mahar D. Wong I. Rune K. (2017). A neurotic extrovert who is open to new experiences? Understanding how personality traits may impact the commenting behaviors of online news readers. J. Broadcast. Electron. Media 61, 557–573. doi: 10.1080/08838151.2017.1344671

[ref14] Beyari H. (2023). The relationship between social media and the increase in mental health problems. Int. J. Environ. Res. Public Health 20:2383. doi: 10.3390/ijerph20032383, 36767749 PMC9915628

[ref15] Bonsaksen T. Thygesen H. Leung J. Lamph G. Kabelenga I. Østertun Geirdal A. (2024). Patterns of social media use across age groups during the COVID-19 pandemic: a study across four countries. Soc. Sci. 13:194. doi: 10.3390/socsci13040194

[ref16] Bowden-Green T. Hinds J. Joinson A. (2020). How is extraversion related to social media use? A literature review. Pers. Individ. Dif. 164:110040. doi: 10.1016/j.paid.2020.110040

[ref17] Carpenter J. M. Green M. C. LaFlam J. (2011). People or profiles: individual differences in online social networking use. Pers. Individ. Dif. 50, 538–541. doi: 10.1016/j.paid.2010.11.006

[ref18] Chamorro-Premuzic T. (2012). Personality and Individual Differences. Glasgow: BPS Blackwell.

[ref19] Chidiac M. Ross C. Marston H. R. Freeman S. (2022). Age and gender perspectives on social media and technology practices during the COVID-19 pandemic. Int. J. Environ. Res. Public Health 19:3969. doi: 10.3390/ijerph192113969, 36360853 PMC9654135

[ref20] Christiaan T. G. Murti H. A. S. (2025). The intersection of personality traits and social media use: implications for adolescent happiness. G-Couns J. Bimbingan Dan Konseling 10, 570–582. doi: 10.31316/g-couns.v10i01.8003

[ref21] Cochran W. G. (1977). Sampling techniques. 3rd Edn New York: John Wiley & Sons.

[ref22] Correa T. Hinsley A. W. De Zúñiga H. (2010). Who interacts on the web? The intersection of users’ personality and social media use. Comput. Hum. Behav. 26, 247–253. doi: 10.1016/j.chb.2009.09.003

[ref23] Costa P. T. McCrae R. R. (1988). Personality in adulthood: a six-year longitudinal study of self-reports and spouse ratings on the NEO personality inventory. J. Pers. Soc. Psychol. 54, 853–863. doi: 10.1037/0022-3514.54.5.853, 3379583

[ref24] Costa P. T. McCrae R. R. (1992). The five-factor model of personality and its relevance to personality disorders. J. Personal. Disord. 6, 343–359. doi: 10.1521/pedi.1992.6.4.343

[ref25] Drążkowski D. Pietrzak S. Mądry L. (2022). Temporary change in personality states among social media users: effects of Instagram use on big five personality states and consumers' need for uniqueness. Curr. Issues Pers. Psychol. 10, 32–38. doi: 10.5114/cipp.2021.110938, 38013757 PMC10653346

[ref26] Đurić P. Ćudina V. Bolić T. Glavak-Tkalić R. (2024). Personality traits, self-esteem, life satisfaction and problematic social media use among young adults. J. Eur. Psychol. Stud. 14, 12–26. doi: 10.59477/jeps.598

[ref27] Dzogbenuku R. K. Doe J. K. Amoako G. K. (2022). Social media information and student performance: the mediating role of hedonic value (entertainment). J. Res. Innov. Teach. Learn. 15, 132–146. doi: 10.1108/JRIT-12-2020-0095

[ref28] Gil de Zúñiga H. Diehl T. Huber B. Liu J. (2017). Personality traits and social media use in 20 countries: how personality relates to frequency of social media use, social media news use, and social media use for social interaction. Cyberpsychol. Behav. Soc. Netw. 20, 540–552. doi: 10.1089/cyber.2017.0295, 28922034

[ref29] Gill A. Nowson S. Oberlander J. (2009). What are they blogging about? Personality, topic and motivation in blogs. Proc. Int. AAAI Conf. Web Soc. Media 3, 18–25. doi: 10.1609/icwsm.v3i1.13949

[ref30] Gmi Blogger Saudi Arabia Social Media Statistics. (2024). Available online at: https://www.globalmediainsight.com/blog/saudi-arabia-social-media-statistics/ (Accessed February 10, 2026).

[ref31] Goby V. P. (2006). Personality and online/offline choices: MBTI profiles and favored communication modes in a Singapore study. Cyberpsychol. Behav. 9, 5–13. doi: 10.1089/cpb.2006.9.5, 16497113

[ref32] Guadagno R. E. Bradley M. O. Cassie A. E. (2008). Who blogs? Personality predictors of blogging. Comput. Hum. Behav. 24, 1993–2004. doi: 10.1016/j.chb.2007.09.001

[ref33] Gupta S. Bashir L. (2018). Social networking usage questionnaire: development and validation in an Indian higher education context. Turk. Online J. Dist. Educ. 19, 214–227. doi: 10.17718/tojde.471918

[ref34] Hama amin D. S. Mohammad P. J. (2024). Social networking usage and mental health problems in the Kurdistan region of Iraq during the COVID-19 outbreak and lockdown. Discov. Psychol. 4:5. doi: 10.1007/s44202-023-00095-1

[ref35] Hamburger Y. A. Ben-Artzi E. (2000). The relationship between extraversion and neuroticism and the different uses of the internet. Comput. Hum. Behav. 16, 441–449. doi: 10.1016/S0747-5632(00)00017-0

[ref36] Huang C. (2022). A meta-analysis of the problematic social media use and mental health. Int. J. Soc. Psychiatry 68, 12–33. doi: 10.1177/0020764020978434, 33295241

[ref37] Ibrahim I. A. Mohamed M. H. M. Alenezi A. (2024). Exploring the linkages between social media use, self-esteem, and academic performance among nursing students in Saudi Arabia: a descriptive correlational study. Belitung Nurs. J. 10, 152–159. doi: 10.33546/bnj.3188, 38690305 PMC11056838

[ref38] John O. P. Srivastava S. (1999). “The big-five trait taxonomy: history, measurement, and theoretical perspectives,” in Handbook of Personality: Theory and Research, eds. Pervin L. A. John O. P.. 2nd ed (New York: Guilford Press), 102–138.

[ref39] Kim Y. Jeong J. S. (2015). Personality predictors for the use of multiple internet functions. Internet Res. 25, 399–415. doi: 10.1108/IntR-11-2013-025

[ref40] Kraut R. Kiesler S. Boneva B. Cummings J. Helgeson V. Crawford A. (2002). Internet paradox revisited. J. Soc. Issues 58, 49–74. doi: 10.1111/1540-4560.00248

[ref41] Kross E. Verduyn P. Sheppes G. Costello C. K. Jonides J. Ybarra O. (2021). Social media and well-being: pitfalls, progress, and next steps. Trends Cogn. Sci. 25, 55–66. doi: 10.1016/j.tics.2020.10.005, 33187873

[ref42] Lin H. Wang C. Sun Y. (2024). How big five personality traits influence information sharing on social media: a meta-analysis. PLoS One 19:e0303770. doi: 10.1371/journal.pone.0303770, 38865331 PMC11168692

[ref43] Manner C. Lane W. (2017). Who posts online customer reviews? The role of sociodemographics and personality traits. J. Consum. Satisfaction Dissatisf. Complain. Behav. 30:23.

[ref45] Marengo D. Poletti I. Settanni M. (2020). The interplay between neuroticism, extraversion, and social media addiction in young adult Facebook users: testing the mediating role of online activity using objective data. Addict. Behav. 102:106150. doi: 10.1016/j.addbeh.2019.106150, 31706139

[ref46] Mark G. Ganzach Y. (2014). Personality and internet usage: a large-scale representative study of young adults. Comput. Hum. Behav. 36, 274–281. doi: 10.1016/j.chb.2014.03.060

[ref47] Masalimova A. R. Kosheleva Y. P. Kosarenko N. N. Kashina S. G. Sokolova E. G. Iakovleva E. V. (2023). Effects of social networking sites on university students’ academic performance: a systematic review. Online J. Commun. Media Technol. 13:e202339. doi: 10.30935/ojcmt/13365

[ref48] McCrae R. R. Costa P. T. Jr. (1997). Personality trait structure as a human universal. Am. Psychol. 52, 509–516. doi: 10.1037//0003-066x.52.5.509 9145021

[ref50] Orben A. (2020). Teenagers, screens and social media: a narrative review of reviews and key studies. Soc. Psychiatry Psychiatr. Epidemiol. 55, 407–414. doi: 10.1007/s00127-019-01825-4, 31925481

[ref51] Özgüven N. Mucan B. (2013). The relationship between personality traits and social media use. Soc. Behav. Pers. 41, 517–528. doi: 10.2224/SBP.2013.41.3.517

[ref52] Pennington C. R. Murray E. A. H. Kaye L. K. Burgess A. P. Kessler K. Shaw D. J. (2025). Social competencies mediate relationships between styles of social media usage and psychosocial wellbeing. Comput. Hum. Behav. Rep. 19:100753. doi: 10.1016/j.chbr.2025.100753

[ref53] Quintelier E. Theocharis Y. (2013). Online political engagement, Facebook, and personality traits. Soc. Sci. Comput. Rev. 31, 280–290. doi: 10.1177/089443931246

[ref54] Rammstedt B. John O. P. (2007). Measuring personality in one minute or less: a 10-item short version of the big five inventory in English and German. J. Res. Pers. 41, 203–212. doi: 10.1016/j.jrp.2006.02.001

[ref55] Roos J. M. (2023). The intersection of personality traits and social media usage: large-scale representative samples of internet users in Sweden. Psych 5, 70–79. doi: 10.3390/psych5010008

[ref56] Roos J. M. Kazemi A. (2021). Personality traits and internet usage across generation cohorts: insights from a nationally representative study. Curr. Psychol. 40, 1287–1297. doi: 10.1007/s12144-018-0033-2

[ref57] Roos J. M. Sprei F. Holmberg U. (2020). Sociodemography, geography, and personality as determinants of car driving and use of public transportation. Behav. Sci. 10:93. doi: 10.3390/bs10060093, 32466504 PMC7349195

[ref58] Ross C. Orr E. S. Sisic M. Arseneault J. M. Simmering M. G. Orr R. R. (2009). Personality and motivations associated with Facebook use. Comput. Hum. Behav. 25, 578–586. doi: 10.1016/j.chb.2008.12.024

[ref59] Ryan T. Xenos S. (2011). Who uses Facebook? An investigation into the relationship between the big five, shyness, narcissism, loneliness, and Facebook usage. Comput. Hum. Behav. 27, 1658–1664. doi: 10.1016/j.chb.2011.02.004

[ref60] Salloum S. A. Al-Emran M. Shaalan K. (2017). Mining text in news channels: a case study from Facebook. Int. J. Inf. Technol. Lang. Stud. 1, 1–9. doi: 10.12785/ijcds/060203

[ref61] Seidman G. (2013). Self-presentation and belonging on Facebook: how personality influences social media use and motivations. Pers. Individ. Dif. 54, 402–407. doi: 10.1016/j.paid.2012.10.009

[ref62] Shaw D. J. Kaye L. K. Ngombe N. Kessler K. Pennington C. R. (2022). It’s not what you do, it’s the way that you do it: an experimental task delineates among passive, reactive and interactive styles of behaviour on social networking sites. PLoS One 17:e0276765. doi: 10.1371/journal.pone.0276765, 36477023 PMC9728879

[ref63] Statista. (2025). Digital population worldwide. Available online at: https://www.statista.com/statistics/617136/digital-population-worldwide/#statisticContainer (Accessed February 10, 2026)

[ref64] Swickert R. J. Hittner J. B. Harris J. L. Herring J. A. (2002). Relationship among internet use, personality and social support. Comput. Hum. Behav. 18, 437–451. doi: 10.1016/S0747-5632(01)00054-1

[ref65] Tandon A. Dhir A. Almugren I. AlNemer G. N. Mäntymäki M. (2021). Fear of missing out (FoMO) among social media users: a systematic literature review, synthesis and framework for future research. Internet Res. 31, 782–821. doi: 10.1108/INTR-11-2019-0455

[ref66] Tarar S. S. Baig F. (2024). Investigating the relationship between personality traits and motivations to use social media among university students in Pakistan. Online J. Commun. Media Technol. 14:e202428. doi: 10.30935/ojcmt/14477

[ref67] Tuten T. Bosnjak M. (2001). Understanding differences in web usage: the role of need for cognition and the five factor model of personality. Soc. Behav. Pers. 29, 391–398. doi: 10.2224/sbp.2001.29.4.391.

[ref68] Wang J. L. Jackson L. A. Wang H. Z. Gaskin J. (2012). The relationship among the big five personality factors, self-esteem, narcissism, and sensation-seeking to Chinese university students’ uses of social networking sites (SNSs). Comput. Hum. Behav. 28, 2313–2319. doi: 10.1016/j.chb.2012.07.001

[ref69] Wolfers L. N. Schneider F. M. (2021). Using media for coping: a scoping review. Commun. Res. 48, 1210–1234. doi: 10.1177/0093650220939778

[ref70] Wu M. Pei Y. (2022). Linking social media overload to health misinformation dissemination: an investigation of the underlying mechanisms. Telemat. Inform. Rep. 8:100020. doi: 10.1016/j.teler.2022.100020

